# Striking variations in consultation rates with general practice reveal family influence

**DOI:** 10.1186/1471-2296-8-4

**Published:** 2007-01-18

**Authors:** Mieke Cardol, Liset van Dijk, Wil JHM van den Bosch, Peter Spreeuwenberg, Dinny H de Bakker, Peter P Groenewegen

**Affiliations:** 1 NIVEL (Netherlands Institute for Health Services Research), Utrecht, The Netherlands; 2 Radboud University, Nijmegen, The Netherlands; 3 Department of Sociology and Department of Human Geography, Utrecht University, The Netherlands

## Abstract

**Background:**

The reasons why patients decide to consult a general practitioner vary enormously. While there may be individual reasons for this variation, the family context has a significant and unique influence upon the frequency of individuals' visits. The objective of this study was to explore which family factors can explain the differences between strikingly high, and correspondingly low, family consultation rates in families with children aged up to 21.

**Methods:**

Data were used from the second Dutch national survey of general practice. This survey extracted from the medical records of 96 practices in the Netherlands, information on all consultations with patients during 2001. We defined, through multilevel analysis, two groups of families. These had respectively, predominantly high, and low, contact frequencies due to a significant family influence upon the frequency of the individual's first contacts. Binomial logistic regression analyses were used to analyse which of the family factors, related to shared circumstances and socialisation conditions, can explain the differences in consultation rates between the two groups of families.

**Results:**

In almost 3% of all families, individual consultation rates decrease significantly due to family influence. In 11% of the families, individual consultation rates significantly increase due to family influence. While taking into account the health status of family members, family factors can explain family consultation rates. These factors include circumstances such as their economic status and number of children, as well as socialisation conditions such as specific health knowledge and family beliefs. The chance of significant low frequencies of contact due to family influences increases significantly with factors such as, paid employment of parents in the health care sector, low expectations of general practitioners' care for minor ailments and a western cultural background.

**Conclusion:**

Family circumstances can easily be identified and will add to the understanding of the health complaints of the individual patient in the consulting room. Family circumstances related to health risks often cannot be changed but they can illuminate the reasons for a visit, and mould strategies for prevention, treatment or recovery. Health beliefs, on the other hand, may be influenced by providing specific knowledge.

## Background

People's decisions to consult a general practitioner vary enormously [1-7]. Next to individual differences that influence consultation rates, the social context of the family has an important and unique influence on individual consultation behaviour. To a certain extent family members resemble each other in their consultation behaviour, even across generations [8]. The association between contact frequencies of mothers and children, especially girls, is well-known [9,10]. Previous studies showed that fathers' frequencies of contact are also associated with those of mothers, and of children [5,11]. Similarity in consultation rates is especially true for families that present with minor ailments such as headache or abdominal pain [11]. So why do members of one family resemble each other in their consultation behaviour? By this behaviour we mean both, in consultation frequencies, as well as health ailments for which a general practitioner is consulted. Explanations must be sought in socialisation and shared family circumstances [12]. Socialisation refers to the internalisation of family beliefs and attitudes related to health and health behaviour. What can be considered illness, and when a visit to a general practitioner is appropriate, are learned within families [8]. Here knowledge is probably influential. A mother's educational level has been shown to be a strong predictor of the frequency of children's new consultations [13]. Shared circumstances refer to a collective social, physical and financial context in which opportunities for health, or threats to health occur. Shared circumstances such as the kind of insurance [14] or family size [15] can explain similarities in consultation rates with general practice among individual family members. With regard to the larger families, time constraints and experience may be of importance. For example, in larger families, parents may have less time to monitor closely the health of all members [15]. On the other hand, earlier research showed that mothers more often consult general practice with their first born than with subsequent children [14]. This may indicate that parents of larger families are more experienced in health care matters, resulting in fewer consultations for all members. Thus, the family context may influence consultation behaviour in such a manner that, for example, from a family viewpoint, the decision not to visit the general practitioner is a logical one. However, from a different viewpoint, a visit may seem appropriate.

Differences between families can help to explain differences in individual consultation rates. If socialisation processes and family circumstances can explain consultation patterns, this would mean that family factors, other than an individual's illness, are part of the decision to consult the general practitioner. Family attitudes towards illness may prevent people from consulting a doctor in time, resulting in unnecessary difficulties for patients or more costs for the health care system. For general practitioners it is interesting to know which family factors influence individual frequencies of contact. This may help both to understand the reasons underlying individual consultations, and to choose adequate strategies for treatment or communication. The question posed in this study is: To what extent can the family context explain differences between both strikingly high, and low, consultation rates among families with children aged up to 21?

## Methods

### Data

Data were used from the second Dutch national survey of general practice [16]. This survey extracted information on all consultations with patients during 2001, from the medical records of 96 practices in the Netherlands (2% of the total number of Dutch practices). These practices were representative of the Netherlands patient population with respect to sex, age, type of health care insurance, and level of urbanisation. The general practitioners in the practices were representative of the Dutch population of general practitioners in most aspects. Only general practitioners working solo were relatively under-represented (31% versus 43%).

Background information about family members' socio-demographic characteristics was collected by means of a one-page questionnaire with 14 items in four languages sent to all people listed with the 96 practices (response: 77%; n = 294 999) [16]. This questionnaire could also be filled in on the internet. A random sample of the Dutch-speaking population of the participating practices took part in an extended health interview at the patients' homes (response 65%; n = 12 699). Respondents of the health interviews were more often female as compared to the Dutch population (54% versus 51%) and more often had a western cultural background (96% versus 91%). Educational level, socio-economic status and self-reported health status were comparable to the Dutch population.

The interview included questions about health complaints, use of health services, attitudes and beliefs. Only one member from each family was interviewed; responses were attributed to all members of that family. In children aged under 12, the interview was conducted with a parent. The internal validity of the one-page questionnaire and the health interview were good [16].

### Dependant variables: realisation of three groups of families

Families were defined as having at least one parent living together with at least one child and being listed in the same practice. Almost all non-institutionalised Dutch citizens are listed in a general practice. Family members are usually listed in the same practice. General practice therefore provides adequate information for studying consultation behaviour in families. For this study we selected families with one or more children aged < = 21 years. The minimum age of children was set at two years because children below this age also attend baby or child health clinics. Families with children below the age of two or above the age of 21 were not excluded. Only the children in the specific age categories were excluded. This resulted in a study population of 42 397 families (Table 1).

**Table 1:** Study data: sources, variables, selections and incomplete information

**Source**	**Study population**	**Selection**	
One year registration of all face-to-face contacts of individuals in electronic medical records	160 926 individuals; 42 397 families	Families with children 2–21 yrs (siblings of 0–2 yrs or >21 yrs within families were excluded)	
One-page questionnaire sent at the patient's home	160 926 individuals; 42 397 families	Families with children 2–21 yrs (siblings of 0–2 yrs or >21 yrs within families were excluded)	
Extended health interview at the patient's home	5 313 individuals aggregated to families	One member of families with children 2–21 yrs	

**Variable view**	**Source**	**Categorisation**	**Incomplete information on family level**

Generation	One-page questionnaire	Parent Child	none
Age	One-page questionnaire	Excluded: families with siblings > 21 yrs (%)	none
Sex	One-page questionnaire	Male Female	none
Family health status	registration in medical record	Consultations for minor ailments: yes/no	none
		Consultations for chronic illness: yes/no	none
	One-page questionnaire	Self-reported health status ranging from 1 (very good) to 5 (very bad)	10 366

*Indicators for shared circumstances*			

Insurance type	One-page questionnaire	Social insurance Private insurance	10 176
Educational level parents	One-page questionnaire	Low/moderate: primary and secondary education High: post secondary and tertiary education	12162
Number of children	One-page questionnaire	Minimum: one parent and one sibling	None
Mother's employment	One-page questionnaire	Paid work yes/no	3626

*Indicators for socialisation*			

Cultural background parents	One-page questionnaire	Both western Heterogeneous Both non-western	10301
Parents' employment in the health care sector	One-page questionnaire	All levels of employment in the health care sector: yes/no	None
Attitude towards management of minor ailments	Health interview	Sumscore on Nijmegen Expectation Questionnaire, range 12–60	2 899
Trust in general practitioners' care	Health interview	One-item question asking for a mark; range 1–10	2608
Self-care activities	Health interview	Conducting self-care in case of minor ailments: yes/no	106

Dependent on the size of the practice, about 152 to 1360 families attended the same practice (median 334). Earlier research into this study population showed that the family accounted for 22% of the variance in the frequency of individuals' first contacts [5].

In the present study we used a multilevel poisson regression model with three levels, individual, family and practice, to define three groups of families based on their family effects [17]. This family effect cannot be measured directly, therefore we extracted it from the frequency of contacts of individual family members. Contact frequency is defined as the number of face-to-face contacts counted for an individual in a year. Only the first contacts in any one period of sickness were chosen because the patients' initiatives are most clear in these contacts. The fixed part of the multilevel model shows the overall mean frequency of contact. Individual consultation rates were predicted on the basis of a parent or child's age and sex, and controlled for clustering of individuals in families, and families in practices. The random part of the model shows the variance on the three levels: individual variance within families; variance between families; and variance between practices. The latter not only captures the influence of general practitioners, but also the geographical contexts of families, because almost all patients live in close proximity to the practice. For this study we used the estimates on the family level, that is family deviation of the overall mean frequency of contact, to define the family effect. This way the family effect consists solely of the unique contribution of families upon the frequency of contact of the individual, corrected for all the other components in the model. The families were then divided into three groups, based on this family effect. The first group of families are called 'lowering-effect families' – families in which members on average have lower contact frequencies than expected based on their age, generation (being a child or parent) and sex. The family effect in this group is statistically significant and negative, which means that family influences cause their frequencies of contact to decrease. The second group of families is composed of those with a significant positive family effect. We call them 'raising-effect families'. In these families, members on average have higher frequencies of contact than one would expect given their age, generation and sex. In these two groups of families the family effect makes a significant difference. The third group consists of average families in which the family effect is associated with both higher and lower frequencies of contact than can be expected on the basis of the individual's characteristics. This third group will only be used as a comparison with the attributes of the families at the extremes (Tables 2 and 3).

**Table 2:** Characteristics of the study population

	**Lowering-effect families**		**Average families**		**Raising-effect families**	
**Individual level**	Parents (n = 2508)	Siblings (n = 3316)	Parents (n = 66863)	Siblings (n = 71114)	Parents (n = 8356)	Siblings (n = 8769)
Mean age (sd; range)	40.5 (6.9; 21–65)	10.5 (5.2; 2–21)	40.4 (7.0; 21–65)	10.5 (5.3; 2–21)	39.4 (7.1; 21–65)	10.3 (5.5; 2–21)
Mean frequency of contact (max)	0.1 (3)	0.1(3)	1.2(9)	0.9(10)	3.5(33)	2.8(17)
% individuals with zero contacts in registration year	88.2	91.4	40.2	48.3	8.2	10.7
% respondents reporting moderate to bad health status^1^	9.1	2.7	12.7	3.8	21.4	6.7

	**Lowering-effect families**		**Average families**		**Raising-effect families**	

**Family level**	n = 1247 families (2.9%)		n = 36 641 families (86.4%)		n = 4509 families (10.6%)	
Mean number of years listed in current practice	12.8		13.5		13.2	
% one parent families	7.9*		20.9		19.1	
Mean number of siblings	3*		2		2	
% privately insured	58.4*		46.7		34	
% one/both parents non-western cultural background	8.7*		9.7		16.1	
% families mothers high educational level	28*		22.5		13	
% families mother paid employment	32*		43.4		37.1	
% families mothers working in health care sector	12.7*		10.1		7.3	
% families fathers working in health care sector	5.9*		2.2		1.2	

^1 ^respondents > = 12 yrs

* differences between lowering-effect and raising-effect families are significant at p < 0.05

**Table 3:** Family health status and attitudes^1 ^towards minor ailments and health care services, by family group

	**Lowering-effect families (n = 1247)**	**Average families (n = 36641)**	**Raising-effect families (n = 509)**
% of families with illness episode(s) of minor ailments for which the GP was consulted	19.9*	67.2	96.0
% of families with illness episode(s) of chronic disease for which the GP was consulted	19.9*	56.0	82.2

	**Lowering-effect families (n = 135)**	**Average families (n = 4604)**	**Raising-effect families (n = 574)**

% respondents using self-care strategies for minor ailments	77.8*	81.8	86.1
Mean sum score Nijmegen Expectation Questionnaire^2^	45.7 *	43.5	42.5
% respondents reporting unsatisfactory marks for trust in health care	23.3	23.0	21.9
% respondents reporting unsatisfactory marks for trust in general practitioner care	11.7	15.3	19.9

^1 ^information from the health care interviews

^2 ^higher scores denote fewer beliefs about the benefits of general practitioner's care for minor health ailments

* differences between families with higher and lower individual contact frequencies are significant at p < 0.05

### Independent variables

#### Health status

Health status is the most important predictor for consultation rates of individuals [1,8]. Health status was evaluated both by individual patients own reports of their health and by the health complaints for which they have consulted their general practitioner. From the medical records we constructed two variables. Firstly, whether family members had consulted general practice for minor ailments such as headache or abdominal pain [18]. And secondly, whether they consulted general practice about a chronic disease [19]. Furthermore, based on the one-tem question of the Short-Form-36 (SF-36; [20]) a mean family score of self-reported health status, ranging from 1 to 5; (very) good to (very) bad, was calculated (Table 1).

#### Shared circumstances

Shared circumstances were defined as: The type of insurance; the educational level of the parents; the employment of the mother; and the number of children (Table 1). The type of insurance was used as a proxy for social economic status. At the time of this study the insurance in the Netherlands was related to patients' income. About two-thirds of the population had obligatory social insurance, whereas people with a higher income had private insurance. In addition, the educational level of the parents was used as an indicator for social status and income. Since the influence of the mother is still dominant, as far as consultation patterns of children are concerned [14], the mother's employment was used as an indicator for time constraints to attend general practice. The number of children in a family refers to time constraints [15] or experience [14].

#### Socialisation

The study design was cross-sectional, which makes it impossible to focus on the *process *of socialisation. Rather, we evaluated socialisation conditions including: the health knowledge of the parents; family health beliefs, such as expectations of general practice care in case of minor ailments; trust in health care; and performance of self-care activities (Table 1). Parents employment in the health care sector was evaluated as an indicator for specific knowledge and networks with knowledge, which is known to affect attitudes and beliefs related to health and health care use [21]. People with a non-western background have a different life-style and different health beliefs as compared to people from a western background, for example regarding eating habits, smoking and the use medication [22,23]. Cultural background was therefore used as a proxy for health beliefs, and divided into 'western', people from industrialised countries, and 'non-western', people from non-industrialised countries.

Attitudes towards the management of minor ailments were evaluated with the validated Nijmegen Expectation Questionnaire as part of the health interview [24]. In this questionnaire respondents' attitudes are ascertained in 12 statements concerning the possible benefits of consulting a general practitioner as compared to self-care. A higher score denotes less belief in the benefits of consulting general practice for common ailments, which means that a person will probably not attend general practice for those complaints. Trust in health care was measured using marks on a scale of one to ten, in which one to five denote unsatisfactory marks. Furthermore, in the health interview, respondents reported whether they opt for self-care when they do not feel well. This gives an impression about their awareness of symptoms and feelings of control over their own health.

### Analyses

Descriptive analyses were used to report on the health status of individual family members, shared socio-economic circumstances, and attitudes towards health and health care for the above mentioned three groups of families. Binomial logistic regression analysis was used to analyse to what extent factors related to socialisation and family circumstances can predict the probability of being a lowering-effect family as compared to a raising-effect family (reference group). The analysis was done twice, once based on the entire population (Table 4), and once based on the respondents of the health interviews (Table 5). The second analysis includes more indicators for socialisation, but the power of the analysis is lower due to a smaller sample size. Therefore, in the analyses presented in Table 5, we omitted all insignificant variables of the final model in Table 4. Both regression analyses were done in three steps to differentiate between the influence of health status, family circumstances and socialisation conditions. Correlations between independent variables did not exceed 0.4.

**Table 4:** Probability of predicting lowering-effect families as compared to raising-effect families; binomial logistic regression analysis in three steps (n = 3156 families)

	**Step 1: health status of family members**		**Step 2: health status of family members + circumstances**		**Step 3: health status of family members + circumstances + socialisation conditions**	
	Odds ratio	Confidence interval	Odds ratio	Confidence interval	Odds Ratio	Confidence interval

*Health status*						
Chronic disease in the family (no = ref)	0.12**	0.09–0.14	0.10**	0.08–0.14	0.10**	0.08–0.13
Family score self-reported health (higher score = more members reported bad health)	0.35**	0.28–0.45	0.44**	0.34–0.56	0.47**	0.37–0.61
*Family circumstances*						
Private insurance (social = reference)			2.36**	1.85–3.01	2.18**	1.71–2.79
Educational level mother (low = ref)			1.28	0.79–2.08	0.88	0.54–1.45
Educational level father (low = ref)			1.87*	1.08–3.23	1.60	0.92–2.78
Number of children			2.25**	1.99–2.53	2.27**	2.01–2.56
Paid employment mother (no = ref)			1.02	0.80–1.30	0.97	0.76–1.23
*Indicators for socialisation*						
Father or mother paid employment in health care sector (not = ref)					2.38**	1.75–3.24
Both parents western cultural background (one or both non-western = ref)					2.51**	1.54–4.10
Nagelkerke R^2^	0.27		0.39		0.42	
Percentage correct	86.1		88.6		88.6	

* significant at the level of p < 0.05;

** significant at the level of p < 0.01

**Table 5:** Probability of predicting lowering-effect families as compared to raising-effect families; binomial logistic regression analysis in three steps (n = 257 respondents of the health interview aggregated to families)

	**Step 1: health status of family members**		**Step 2: health status of family members + circumstances**		**Step 3: health status of family members + circumstances + socialisation conditions**	
	Odds ratio	Confidence interval	Odds ratio	Confidence interval	Odds Ratio	Confidence interval

*Health status*						
Chronic disease in the family (no = ref)	0.13**	0.06–0.30	0.13**	0.04–0.27	0.14**	0.05–0.38
Family score self-reported health (higher score = more members reported bad health)	0.19**	0.08–0.46	0.30*	0.12–0.87	0.38	0.14–1.03
*Family circumstances*						
Private insurance (social = reference)			2.63*	1.07–6.42	3.01*	1.11–8.13
Number of children			2.40**	1.59–3.64	2.47**	1.57–3.88
*Indicators for socialisation*						
Father or mother paid employment in health care sector (not = ref)					2.40	0.82–7.01
Both parents western cultural background (one or both non-western = ref)					0.37	0.07–2.11
Sum score Nijmegen expectation Questionnaire^1^					1.14*	1.04–1.24
Self-care in minor ailments					0.54	0.19–1.54
Not much trust in GPs (much = ref)					0.23	0.04–1.22
Nagelkerke R^2^	0.30		0.43		0.51	
Percentage correct	87.9		87.9		89.5	

^1 ^a higher score denotes lower expectations

* significant at the level of p < 0.05;

** significant at the level of p < 0.01

## Results

In almost 3% of all families, the family effect on individual consultation rates was significantly negative (lowering-effect families), in 11% of the families the family effect was significantly positive: these are the raising-effect families. Based on the multilevel analyses, the average effect of lowering-effect families on the frequencies of individuals first contacts is -0.5 contacts per year, per individual (95% CI: -0.9 to -0.3;). In these families, the contact frequency per year for each family member is 0.5 lower than can be expected on the basis of age and sex. For the raising-effect families this figure is +1.6 per year, per individual (95% CI: +0.5 to +5.3).

As expected, in raising-effect families more members rate their health as bad (Table 2). Furthermore, lowering-effect families on average have more children, a private insurance and the parents more often have a higher educational level. Parents in these families more often work in the health care sector. The health interviews show that respondents from lowering-effect families have fewer expectations of general practitioners' care in cases of minor ailments than respondents from raising-effect families (Table 3). The amount of trust in general practitioner care does not differ significantly between the family groups.

In the logistic regression analysis we analysed to what extent health status, shared family circumstances and socialisation conditions can explain differences in consultation behaviour between lowering-effect and raising-effect families (Table 4). In step one we included family health status variables. The chance of low frequencies of contact due to a family effect is reduced by an episode of chronic illness suffered by one of the family members, but also, though to a lesser extent, by family members reporting themselves as having a worse health status. In step two, variables referring to shared circumstances were entered into the analysis. It shows that a higher socio-economic status, as indicated by private insurance, and to a lesser extent the fathers' educational level, both increase the probability of a lowering-effect family. The effect of more siblings in the family is as strong as the effect of being privately insured. Whether the mother has paid employment does not make a significant difference.

In step three, socialisation conditions are added to the model. Paid employment of one of the parents in the health care sector, and both parents having a western cultural background, significantly increase the chance of low frequencies of contact due to a family effect. When adding the indicators for socialisation, the effect of the fathers' educational level is no longer significant. In Table 5 other socialisation conditions, based on the health interview, have been added to the regression model. By doing this, employment in the health care sector and cultural background no longer explain differences between lowering-effect and raising-effect families. Expectations of doctor's care in case of minor ailments do explain part of the differences between lowering-effect and raising-effect families. In the final model, in both analyses, the percentage of correct classification is almost 90%.

## Discussion

This research focuses on the family influence upon an individual's consultation behaviour. The results indicate that striking variations in family consultation rates can be explained, especially by the family health status, but also by family circumstances such as economic status and number of children, as well as socialisation factors such as health knowledge and family beliefs. This study is unique in that the effect of the family has been translated into higher or lower individual contact frequencies than could be expected on the basis of an individual's characteristics. For example, per individual, per year, members of lowering-effect families have 0.5 fewer first contacts than could be expected, based upon their individual characteristics. This may not seem much, but it is substantial in view of the individual mean frequency of first contacts: 1 contact for each member per year.

It must be noted that in this study we evaluated the family effect on frequencies of contact in general. Related to specific diagnoses the family effect on frequencies of contact may show other relationships.

### Results in view of the literature and general practice

The family health status explains most variance in consultation behaviour between the families. This corresponds with the existing literature on individual consultation behaviour [1]. However, the health status of individual family members alone cannot sufficiently explain why in some families, members consult more often than in other families [25]. Family health is thought to be more than the sum of its parts; it connotes the functioning of the family as a primary agent in the promotion of health and well-being [8].

In addition to family health status, another consideration for general practitioners is whether a patient belongs to a larger family, since this decreases the odds for high consultation rates due to family influence. As stated in the introduction, a lack of time [15] or the parents greater experience [14] in health care matters are suggested as other possible explanations. The results of this study point towards experience as an explanation, since the paid employment of mothers, as an indicator for time constraints, did not make a significant difference. Yet another explanation is that in families in which the parents or a first child are not healthy, fewer children are born. As such, the explanation would refer to a selection of more healthy individuals in larger families.

Indeed, knowledge and the family's health beliefs, influence people's use of health services alongside other considerations such as the family health status and shared circumstances [26]. The family is the first context where this is learned, which makes it all the more important for general practitioners to be informed about the patients' social context. Parents paid employment in health care partly explains why families consult less. In those families members have fewer expectations of general practitioners' care for minor ailments and they probably have more specific knowledge and useful networks [21]. Apparently, specific knowledge has more impact than a high educational level. We cannot draw conclusions on the basis of this study about the appropriateness of the contacts with general practice. However this could mean that in families in which the family influence causes consultation rates to increase, it would help to provide more specific knowledge. For example to inform family members about the background and treatment of minor ailments and about when one needs to be concerned about one's health. The results also show that families with more specific knowledge are no more likely to opt for more self-care. Apparently, self-care does not substitute for practice visits. Other studies show that more self-care is associated with more contacts with the practice [21]. Maybe the specific knowledge of health care workers results in a more stoic attitude towards health ailments: they have seen people with poorer health conditions.

The family context is a factor general practitioners can identify in the consultation room that will add to their understanding of the health complaints of the individual patient. To do so, easy and feasible questions have been developed for use in consultations to enable the general practitioner to be informed about the family context of individual patients [2,3,27].

### Limitations of the study

The cross-sectional design of the study made it difficult to evaluate socialisation processes. Instead, we made use of indicators to evaluate socialisation conditions and socio-economic family circumstances. Our indicators were based, as much as possible, on existing knowledge, but they may still have caused bias. Also, the questions asked in the health interview were not directed at family functioning and family relationships, known to be of importance for outcomes related to health and consultation patterns [3].

Furthermore, the relatively small sample that completed the health interviews restricted the possibilities and power of the second regression analysis. We had to eliminate the non-significant variables from the analyses to keep a stable model. To limit the risk of bias this may have caused, we analysed the omitted variables in separate logistic analyses, controlled for the family health status. By doing this, only the educational level of the father was significant (p = 0.01). However, accounting for the educational level of the father in the final analysis did not produce different results. But this may also be caused by the issue of missing data. Information about the type of insurance, the educational level of the parents and the cultural background of the parents was missing in about 20% of the families. Such incomplete information about the educational level of the parents and cultural background, occurred significantly more often in the families with strikingly low consultation rates.

## Conclusion

This study shows that general practitioners need to take the social context of patients into account. Information about their patients' social contexts helps general practitioners to add or delete probabilities that may point towards correct diagnoses and treatment.

Next to the family health status, shared circumstances and socialisation play an important role. Such factors as a high income, larger family size, specific health knowledge, health beliefs, and expectations of care, explain part of the differences in contact frequencies between families with low and families with high consultation rates, due to a family effect. Specific knowledge and/or networks in the health care sector seem more influential than the educational level of the parents. Socio-economic circumstances often cannot be changed but may shed a different light on prevention, treatment or recovery. In conclusion, for general practitioners, it seems fruitful to stay alert to factors beyond individual illness and to make the extra benefits of such a contextual approach more visible. In the Netherlands, they are the only health practitioners that can take advantage of the fact that they often see members of the same family in their consulting room.

## Competing interests

The author(s) declare that they have no competing interests.

## Authors' contributions

MC, LvD, WvdB, DdeB and PG were involved in the study design. Primary data-analysis was conducted by PS and MC, with the other authors contributing. MC drafted the paper, which was revised by all co-authors. All authors read and approved the final manuscript.

## Pre-publication history

The pre-publication history for this paper can be accessed here:


